# High-temperature GC-MS-based serum cholesterol signatures may reveal sex differences in vasospastic angina[S]

**DOI:** 10.1194/jlr.D040790

**Published:** 2014-01

**Authors:** Hyun-Hwa Son, Ju-Yeon Moon, Hong Seog Seo, Hyun Hee Kim, Bong Chul Chung, Man Ho Choi

**Affiliations:** * Future Convergence Research Division, Korea Institute of Science and Technology, Seoul 136-791, Korea; † Cardiovascular Center, Korea University Guro Hospital, Seoul 152-703, Korea

**Keywords:** hybrid solid-phase extraction-precipitation, cardiovascular disease, hydroxycholesterol

## Abstract

Alterations of cholesterol metabolism are responsible for vasospastic angina and atherosclerosis. To comprehensively evaluate cholesterol metabolism, 18 sterols, including cholesterol, 6 cholesteryl esters (CEs), 3 cholesterol precursors, and 8 hydroxycholesterols (OHCs), were simultaneously analyzed using hybrid solid-phase extraction (SPE) purification coupled to high-temperature gas chromatography-mass spectrometry (HTGC-MS). Methanol-based hybrid SPE increased the selective extraction, and HTGC resulted in a good chromatographic resolution for the separation of lipophilic compounds. The limits of quantification of cholesterol and CEs ranged from 0.2 to 10.0 μg/ml, while OHCs and cholesterol precursors ranged from 0.01 to 0.10 μg/ml. Linearity as the correlation coefficient was higher than 0.99 with the exception of cholesteryl laurate, myristate, oleate, and linoleate (*r*^2^ > 0.98). The precision (% coefficient of variation) and accuracy (% bias) ranged from 1.1 to 9.8% and from 75.9 to 125.1%, respectively. The overall recoveries of CEs ranged from 26.1 to 64.0%, and the recoveries of other sterols ranged from 83.8 to 129.3%. The cholesterol signatures showed sex differences in patients with vasospastic angina and may associate with 24-reductases. This technique can be useful for making clinical diagnoses and for an increased understanding of the pathophysiology of vasospastic angina.

Cholesterol is an amphipathic lipid that is an essential component of cell membranes (1), and it is a precursor for conversion to other steroids in the body, such as progestins, sex hormones, and corticosteroids. Its biosynthesis is started from acetyl-CoA (2), which leads to the formation of lanosterol, the first cholesterol precursor having a steroidal skeleton, and is further metabolized in two alternate pathways. One pathway includes lathosterol and 7-dehydrocholesterol as cholesterol precursors, and desmosterol is the precursor in the other pathway.

The cholesterol metabolism in tissues and blood can be described as follows. First, acyl-CoA:cholesterol acyltransferase (ACAT) and lecithin:cholesterol acyltransferase (LCAT) are involved in the conversion to cholesteryl esters (CEs) with fatty acids (3, 4). CEs act as a major form of transporter as plasma lipoproteins, or as storage units as lipid droplets (5). Second, cholesterol is converted to hydroxycholesterols (OHCs) by enzymatic (hydroxylase, cytochrome P450 families) or nonenzymatic hydroxylations at various positions (6). OHCs are well-known as regulators of cholesterol homeostasis (7, 8) (**Fig. 1**).

**Fig. 1.**
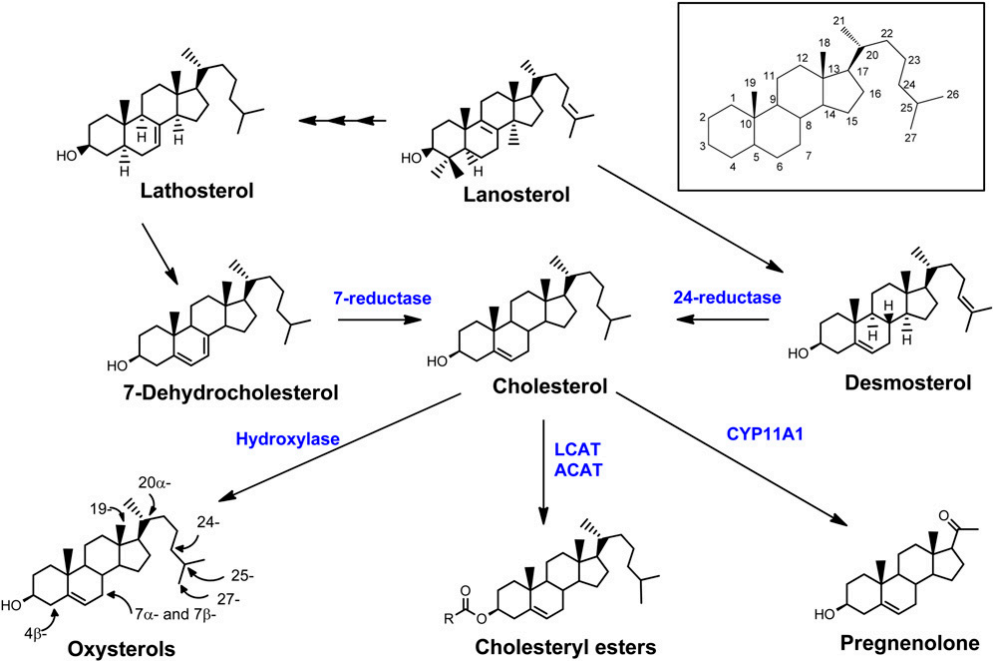
An overview of cholesterol metabolism in humans. Cholesterol biosynthesis is initiated from lanosterol; lanosterol is converted to cholesterol through desmosterol, lathosterol, and 7-dehydrocholesterol as intermediates. 24-Reductase and 7-reductase are enzymes for the production of cholesterol from desmosterol and 7-dehydrocholesterol, respectively. Cholesterol forms CEs with fatty acid by LCAT and ACAT, and it forms OHCs by enzymatic (hydroxylase) or nonenzymatic hydroxylation at the C-4, C-7, C-19, C-20, C-24, C-25, and C-27 positions (see inset).

Alterations of cholesterol metabolism are associated with vasospastic angina, which is considered an early manifestation of atherosclerotic vascular disease. The elevated level of cholesterol is a diagnostic feature of cardiovascular events, and the increased synthesis of CEs is also associated with atherosclerosis (9). In addition, OHCs are linked to a risk of atherosclerosis (10). In particular, 27-OHC inhibits cardioprotective effects of estrogen by competitive antagonism of estrogen receptor binding in the vasculature (11), and a high plasma level of 7β-OHC was observed in a population with a high risk for coronary heart disease (12). Therefore, the comprehensive profiling of cholesterol and related sterols, including CEs, cholesterol precursors, and OHCs, is needed to understand the physiologies of cardiovascular events.

Reliable analytical methods have been described for the quantification of cholesterol (13), CEs (14, 15), OHCs (16, 17), cholesterol precursors, and other related sterols (18, 19) using gas chromatography (GC) or liquid chromatography (LC) coupled to mass spectrometry (MS). Due to the complex regulatory network in cholesterol metabolism, the various types of cholesterols should be estimated accurately and simultaneously in biological specimens. Here, we describe the quantitative cholesterol signatures in the simultaneous analysis of 18 endogenous sterols, including cholesterol, 6 CEs, 3 cholesterol precursors, and 8 OHCs. This analysis uses hybrid solid-phase extraction (SPE)-precipitation coupled to high-temperature GC-MS (HTGC-MS) with diminished matrix backgrounds and improved chromatographic properties of the lipophilic analytes. Subsequently, the devised method was applied to human serum samples that were obtained from patients with vasospastic angina.

## MATERIALS AND METHODS

### Chemicals

The reference standards of 18 analytes examined in this study (**Table 1**) were obtained from Sigma (St. Louis, MO). The deuterium-labeled internal standards (ISs) used were: 2,2,3,4,4,6-*d*_6_-cholesterol and 2,2,3,4,4,6-*d*_6_-cholesteryl stearate (C/D/N Isotopes, Pointe-Claire, Quebec, Canada) for cholesterol and CEs, respectively; and 25,26,26,26,27,27,27-*d*_7_-4β-OHC and 25,26,26,26,27,27-*d*_6_-27-OHC (Avanti Polar Lipids, Alabaster, AL) for three cholesterol precursors and eight OHCs. The trimethylsilylating (TMS) agents, *N*-methyl-*N*-trifluorotrimethylsilyl acetamide (MSTFA), ammonium iodide (NH_4_I), and dithioerythritol (DTE), were purchased from Sigma. The hybrid SPE-precipitation cartridge (H-PPT) (1 ml, 30 mg) was supplied by Supelco (Bellefonte, PA). All organic solvents were of analytical and HPLC grade and were purchased from Burdick and Jackson (Muskegon, MI).

**TABLE 1.** The GC-MS information for the quantitative analysis of 18 sterols analyzed

Compounds	MW*^a^*	Number of TMS*^b^*	Characteristic Ions	Quantitative Ions	Retention Time (min)
Cholesterol	458	1	458, 368, 353, 329, 129	368 [M–OTMS]^+^	4.82
CEs					
Cholesteryl laurate	568	0	368, 353, 247	368 [M–ROOH]^+^	12.40
Cholesteryl myristate	596	0	368, 353, 247	368 [M–ROOH]^+^	14.40
Cholesteryl palmitate	624	0	368, 353, 247	368 [M–ROOH]^+^	17.28
Cholesteryl linoleate	648	0	368, 353, 247	368 [M–ROOH]^+^	20.52
Cholesteryl oleate	650	0	368, 353, 247	368 [M–ROOH]^+^	20.52
Cholesteryl stearate	652	0	368, 353, 247	368 [M–ROOH]^+^	20.73
Cholesterol precursors					
Desmosterol	456	1	456, 441, 366, 343, 327, 129	343 [M–C_8_H_17_]^+^	5.02
Lathosterol	458	1	458, 443, 353, 255	458 [M]^+^	5.16
Lanosterol	498	1	498, 393	393 [M–OTMS–CH_3_]^+^	5.97
OHCs					
7-KC	546	2	546, 456	456 [M–OTMS]^+^	5.80
7β-OHC	546	2	546, 456, 366	456 [M–OTMS]^+^	5.52
4β-OHC	546	2	546, 456, 441, 417, 366, 327, 147	366 [M–2OTMS]^+^	5.75
20α-OHC	546	2	461, 281, 201	201 [TMS-O^+^=C_8_H_16_]^+^	6.05
27-OHC	546	2	546, 456, 441, 417, 129	417 [M–TMS-O^+^=CHCH=CH_2_]^+^	6.90
24S-OHC	546	2	546, 503, 456, 413, 159, 145, 129	413 [M–OTMS–CH(CH_3_)_2_]^+^	6.55
19-OHC	546	2	456, 366, 353	353 [M–OTMS–TMS-O^+^=CH_2_]^+^	5.30
25-OHC	546	2	456, 366, 327, 271, 131	456 [M–OTMS]^+^	6.65

MW, molecular weight (amu).

a Molecular weight as the TMS derivatives.

b The number of TMS-substituted groups.

### Calibration and quality control samples

Each stock solution of cholesterol, cholesterol precursors, and OHCs was prepared at a concentration of 1 mg/ml in methanol, whereas CEs were made up with methanol/chloroform (1:1, v/v) because they were insoluble in absolute methanol. The mixed working solution of three cholesterol precursors and eight OHCs was diluted with methanol at various concentrations ranging from 0.01 to 5 μg/ml, and another mixed working solution, including cholesterol and six CEs, was prepared at 12 different concentrations ranging from 0.2 to 1,000 μg/ml in methanol/chloroform (1:1, v/v). All standard solutions were stored at 4°C until use. For preparing both calibration and quality control (QC) samples, the commercially available steroid-free serum (Scipac, Sittingbourne, UK) was used after checking for all analytes with negative results. Charcoal-stripping is often needed if the commercial sample contains cholesterol in trace.

### Subjects and sample collection

The human serum samples were obtained from 65 male (age 55.2 ± 11.5 years, mean ± SD) and 89 female (age 53.5 ± 14.8 years) patients with vasospastic angina at the Cardiovascular Center of the Korea University College of Medicine (Seoul, Korea). To diagnose or exclude coronary artery disease, coronary angiography was performed on 154 patients who had chest pain typical or atypical of angina pectoris. The patients in this group who had less than 30% fixed stenosis on quantitative coronary angiography were given the acetylcholine provocation test. Acetylcholine was injected into the left coronary artery using incremental doses of 20 mg, 50 mg, and 100 mg for 20 s with at least 3 min between injections. Vasospastic angina was diagnosed when typical chest pain occurred with intracoronary acetylcholine injection or when significant vasoconstriction (>70% stenosis) occurred. The experimental protocol (KUGH-12118) was approved by the Institutional Review Board Committee of the Human Research Protection Center at the Korea University School of Medicine, and an informed consent form was signed by all subjects in compliance with the Declaration of Helsinki principles.

### Sample pretreatment

The serum samples (20 μl) spiked with 20 μl of the IS mixtures (*d*_6_-cholesterol and *d*_6_-cholesteryl stearate, 100 μg/ml; *d*_7_-4β-OHC and *d*_6_-27-OHC, 2 ng/ml) were added to 0.5 ml methanol. The mixture was vortexed for 5 min and centrifuged for 2 min at 12,000 rpm for protein precipitation. The samples were loaded into H-PPT cartridges and eluted three times with 0.5 ml of methanol. The collected eluates were evaporated to remove methanol using an N_2_ evaporator at 40°C and dried in a vacuum desiccator over P_2_O_5_/KOH for at least 30 min. Finally, the dried residues were derivatized in 40 μl of MSTFA/NH_4_I/DTE (500:4:2, v/w/w) for 20 min at 60°C, and 2 μl of the resulting mixture was injected for GC-MS analysis in the selected-ion monitoring (SIM) mode.

### Instrumental conditions

GC-MS was performed with an Agilent 6890 Plus gas chromatograph interfaced with a single-quadrupole Agilent 5975C MSD (Agilent Technologies, Palo Alto, CA). The electron energy was 70 eV and the ion source temperature was 230°C. Each sample (2 μl) was injected in split mode (10:1) at 280°C and separated through a MXT-1 cross-linked dimethylpolysiloxane capillary column (30 m × 0.25 mm inner diameter, 0.25 μm film thickness, Silcosteel-treated stainless steel; Restek, Bellefonte, PA). The oven temperature was held initially at 260°C for 3 min, ramped to 320°C at 10°C/min, increased to 330°C at 2°C/min (held for 8 min), and finally increased to 380°C at 30°C/min and held for 3 min. The carrier gas was ultra-high-purity helium at a column head pressure of 75.8 kPa (14.2 psi; column flow: 1.1 ml/min at an oven temperature of 260°C). For quantitative analysis, the characteristic ions of each compound were determined as their TMS derivatives. Peak identification was achieved by comparing the retention time and matching the height ratios of the characteristic ions (Table 1).

### Method validation

The QC samples containing 18 sterols were quantified by MS peak height ratios versus the IS. The calibration samples were prepared at 16 different concentrations depending on the sensitivity and reference value of the analytes in human serum. Least-squares regression analysis was performed for peak height ratios at increasing analyte levels to obtain calibration linearity. The limits of quantification (LOQs) were defined as the lowest concentrations by conducting five replicate analyses with a precision of 20% and accuracy between 80 and 120%. The precision and accuracy were expressed as the coefficients of variation (% CV) and percent relative error (% bias), respectively, and were determined using QC samples at three different concentrations (low, 1–200 μg/ml; medium, 5–500 μg/ml; and high, 15–750 μg/ml for cholesterol and CEs; low, 0.1 and 0.35 μg/ml; medium, 0.35 and 1.5 μg/ml; and high, 0.75 and 3.5 μg/ml for cholesterol precursors and OHCs) based on the individual analyte calibration ranges. To determine the same-day repeatability, five replicates were analyzed, whereas day-to-day reproducibility was performed by repeating the measurements on five different days. The extraction recovery was established using QC samples at three concentrations in triplicate for each analyte by adding known amounts of mixed working solutions to free serum samples. Absolute recoveries were calculated by comparing peak height ratios of extracted samples versus those of standard samples without sample preparation to represent 100% recovery.

The stability of the analyte during sample collection and handling, which is a prerequisite of reliable quantification, was evaluated. The stability was measured by comparing the results of the samples analyzed before and after being exposed to the conditions for the stability assessment at three different concentrations in triplicate. First, the stability of the standard solutions was tested by allowing them to stand at room temperature for 6 h over the time required for sample preparation. Second, the freeze-thaw stability was determined after three freeze-thaw cycles. After storing three aliquots of QC samples at −20°C for 24 h, the samples were thawed at room temperature. When thawed completely, the samples were refrozen for 12 h under the same conditions, and these processes were repeated three times. Third, the short-term temperature stability was evaluated by thawing the QC samples at ambient temperature and then leaving them to stand at this temperature for 6 h. Fourth, the post-preparative stability was evaluated by reinjecting the prepared samples after 12 h (after one batch analysis of validation samples) and 24 h (1 day after samples were placed in the auto-injector sample tray).

### Statistical analysis and steroid signatures

Data manipulation was performed with Sigmaplot (SYSTAT Software Inc., San Jose, CA). Quantitative results are expressed as the mean ± standard deviation (SD), and group comparisons were made using an unpaired two-tailed Student's *t*-test. *P* < 0.05 was considered statistically significant.

## RESULTS

### GC-MS

**Fig. 2.**
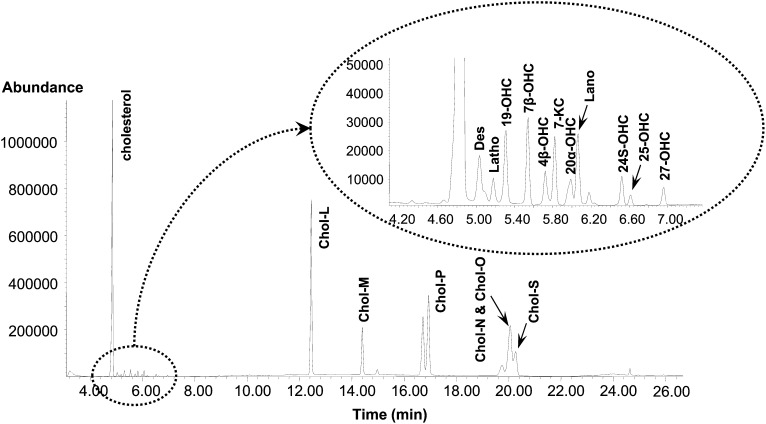
The total ion chromatogram of 18 sterols analyzed by HTGC-MS analysis in the selected-ion monitoring mode of 18 sterols including cholesterol, 6 CEs, 3 cholesterol precursors, and 8 oxysterols. The oven temperature was held initially at 260°C for 3 min, ramped to 320°C at 10°C/min, increased to 330°C at 2°C/min (held for 8 min), and finally increased to 380°C at 30°C/min and then held for 3 min. Des, desmosterol; Latho, lathosterol; Lano, lanosterol; Chol-L, cholesteryl laurate; Chol-M, cholesteryl myristate; Chol-P, cholesteryl palmitate; Chol-N, cholesteryl linoleate; Chol-O, cholesteryl oleate; Chol-S, cholesteryl stearate.

For the profiling of 18 sterols, including cholesterol, 6 CEs, 3 cholesterol precursors, and 8 OHCs, H-PPT purification and HTGC-MS using a thermally stable stainless steel MXT-1 capillary column were conducted. H-PPT was used to remove matrix backgrounds such as phospholipids in the biological samples (20), and the HTGC technique was used to separate high molecular weight and lipophilic compounds (21). All analytes were successfully separated and detected without any interference within a 27 min chromatographic run. Cholesterol, three cholesterol precursors, and eight OHCs were eluted within 7 min, while six CEs were eluted in the order of the number of carbons in the hydrocarbon chain: cholesteryl laurate (CE 12:0), myristate (CE 14:0), palmitate (CE 16:0), oleate (CE 18:1), linoleate (CE 18:2), and stearate (CE 18:0) (**Fig. 2**). It was not easy to separate cholesteryl oleate, linoleate, and stearate because of the same 18-hydrocarbon chain as the fatty acid moiety, but the present chromatographic conditions could selectively quantify cholesteryl stearate from the coeluates cholesteryl oleate and linoleate.

Cholesterol and cholesterol precursors have a hydroxyl group at the C-3 position, and OHCs have two polar functional groups: one is a hydroxyl group at the C-3 position and the other is a hydroxyl or keto group at the C-4, C-7, C-19, C-20, C-24, C-25, or C-27 positions (Fig. 1). In TMS derivatization, both hydroxyl and carbonyl ketone groups were derivatized with TMS, while CEs were unaffected by TMS agents because they do not have polar groups in their chemical structures (Fig. 1, Table 1). In general, 7-ketocholesterol (7-KC) should be 2 Da lighter than equivalent 7-OHC, but it could be easily reduced to produce molecular ions at *m/z* 546 under the present derivatization conditions. To confirm the reduction of 7-KC during the present derivatization, an alternative trimethylsilylation with MSTFA/1% trimethylsilylchloride (TMCS) was performed. In the MSTFA/TMCS reaction, 7β-OHC produced a 3,7-di-TMS derivative, while 7-KC was derivatized at only the C-3 position.

The characteristic ions of cholesterol were observed at *m/z* 458 [M]^+^, *m/z* 443 [M–15; M–CH_3_]^+^, *m/z* 368 [M–90; M–OTMS]^+^, *m/z* 353 [M–90–15; M–OTMS–CH_3_]^+^, *m/z* 329 [M–129; M–TMS-O^+^=CHCH=CH_2_]^+^, and *m/z* 129 [TMS-O^+^=CHCH=CH_2_]^+^, which are in accordance with a general mass spectral interpretation (22). Among these fragments, the *m/z* 368 ion was chosen as the quantitative ion. All CEs generated a base peak at *m/z* 368 by cleavage of the ester bond, regardless of the fatty acid moiety (23). The quantitative ion of desmosterol was selected to be the *m/z* 343 ion that was formed by the loss of the side chain and two nuclear hydrogens. The quantitative ions of lathosterol and lanosterol were selected to be *m/z* 458 [M]^+^ and *m/z* 393 [M–90–15]^+^, respectively. In addition, OHCs showed different fragmentation patterns depending on the –OH positions (Table 1) (24). These results may provide useful information about the chemical structures of cholesterol and its metabolites.

### Method validation

Method validation requires an evaluation of LOQ, linearity, accuracy, and precision using calibration and QC samples prepared from cholesterol and related sterol-free serum. The LOQs of cholesterol and CEs ranged from 0.2 to 10.0 μg/ml. The LOQs of cholesterol precursors and OHCs were 0.01–0.10 μg/ml. The calibration curve consisted of a blank sample and 15 different points ranging from the LOQ to the expected concentration in the sample. The devised method showed excellent linearity with the correlation coefficient (*r*^2^ > 0.99) for all compounds except cholesteryl laurate, myristate, oleate, and linoleate (*r*^2^ > 0.98). Accuracy and precision were determined by analyzing the QC samples at three different concentrations (low, medium, and high) according to the calibration range. With respect to repeatability, intra-day (n = 5) precision (% CV) ranged from 1.1 to 10.9%, while accuracy (% bias) ranged from 75.9 to 125.1%. As reproducibility, inter-day (n = 5) precision and accuracy were 3.0–9.3% and 76.5–122.9%, respectively. The overall recoveries of CEs ranged from 26.1 to 64.0%, and the recoveries of other sterols ranged from 83.8 to 129.3%. Although CE recoveries were low, quantification was possible because the results were reproducible and accurate (supplementary Table I).

The stability tests were evaluated for the reliable quantification of analytes under several conditions, including standard solution storage, short-term storage (bench top, room temperature), freeze and thaw cycles, and the analytical process as described in the Materials and Methods section. Both stock solutions and QC samples were prepared fresh, and the standard solutions were stable at −20°C for 3 months and at room temperature for 6 h. The short-term stability, which was tested by thawing the QC samples at 25°C and leaving them to stand for 6 h, showed no significant changes (>80% recovery) in concentration under the conditions tested, except for CEs (24.6–82.9% yields) (supplementary Table II). The freeze/thaw stability was evaluated from the concentration of an aliquot that was not subjected to freeze/thaw cycles as a reference. The stability of the cholesterols was also demonstrated in serum samples subjected to three freeze/thaw cycles. The overall differences between the cycles were not significant within a 15% deviation in most conditions, except for CEs. Repeating the freeze/thaw cycles did not appear to affect the concentration of analytes. Because instability can occur not only in the sample matrix but also in the prepared samples, it is important to test the post-preparative stability under the conditions of analysis, including the autosampler conditions, for the expected maximum time of an analytical run to determine if the analytical run could be reanalyzed in the case of instrumental failure. Our results showed that the analytes were quite stable when the prepared samples were injected 1 day after being placed in the sample tray >85% recovery.

### Application to human serum with vasospastic angina

The usefulness of this method was demonstrated with serum samples obtained from 154 patients with vasospastic angina. All quantitative results were compared between 65 male and 89 female patients using the Student's *t*-test, and the concentrations were expressed as μg/ml for cholesterol and CEs and as ng/ml for cholesterol precursors and OHCs (**Table 2**). Among 18 compounds, 7-KC, 20α-, 19-, and 25-OHC were not detected in most samples, while 24S-OHC was detected in a few serum samples **(**Table 2, supplementary Fig. I**)**. Serum concentrations of cholesterol, CEs, and OHCs were not significantly different in both sexes. However, the level of lathosterol, a cholesterol precursor, was higher in the male patients than in the female patients (1,608.0 ± 1,115.4 ng/ml vs. 1,240.1 ± 837.7 ng/ml; *P* < 0.03).

**TABLE 2.** Concentration of 12 sterols detected in human serum obtained from patients with vasospastic angina

Compounds	Concentration*^a^*		*P*
	Males*^b^*	Females*^c^*	
Cholesterol	395.2 ± 121.3	413.9 ± 135.4	0.37
CEs			
Cholesteryl laurate	0.7 ± 0.4	0.7 ± 0.5	0.97
Cholesteryl myristate	36.7 ± 21.2	41.4 ± 27.6	0.24
Cholesteryl palmitate	144.1 ± 43.4	154.9 ± 47.7	0.15
Cholesteryl oleate and linoleate	445.0 ± 127.8	469.1 ± 142.2	0.27
Cholesterol precursors			
Desmosterol	349.1 ± 144.8	307.4 ± 180.5	0.11
Lathosterol	1608.0 ± 1115.4	1240.1 ± 837.7	0.03
Lanosterol	150.5 ± 93.5	132.1 ± 96.1	0.23
OHCs			
7β-OHC	57.0 ± 33.6	57.9 ± 34.1	0.88
4β-OHC	23.7 ± 6.7	25.7 ± 10.1	0.15
27-OHC	25.8 ± 9.8	22.6 ± 9.5	0.05
24S-OHC	Trace	Trace	—

a Concentrations are expressed as μg/ml for cholesterol and CEs, and as ng/ml for cholesterol precursors and OHCs.

b Males: n = 65; age 55.20 ± 11.52 years.

c Females: n = 89; age 53.48 ± 14.76 years.

**Fig. 3.**
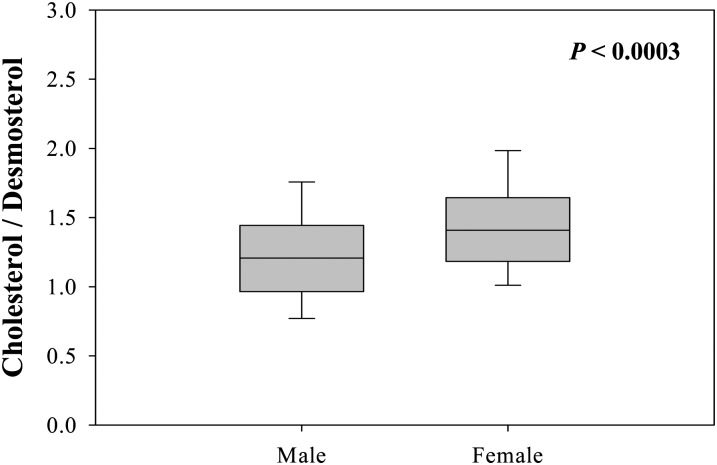
Comparison of 24-reductase activity in male and female patients with vasospastic angina. The enzyme activity of 24-reductase was expressed as metabolic ratios of cholesterol to desmosterol.

The quantitative results obtained from this method can also be expressed as enzyme activities using the metabolic ratio of metabolite to precursor. The ratio of cholesterol to desmosterol (24-reductase; *P* < 0.0003) was significantly higher in the female patients than in the male patients (**Fig. 3**). This suggests that cholesterol synthesis in female patients with vasospastic angina is more activated than in males.

## DISCUSSION

An alteration in cholesterol metabolism is a powerful predictor of developing cardiovascular events, even in an early stage of atherosclerosis that is associated with coronary spasm. Many studies focus on partial measurements of the cholesterol metabolites (13–19), but this is not enough to monitor the overall cholesterol metabolism. Therefore, we have developed cholesterol signatures, which are quantitative profiles of cholesterol, CEs, cholesterol precursors, and OHCs.

Several chromatographic methods have been described for the measurement of cholesterol and related sterols. The LC-MS-based analytical techniques have shown excellent sensitivity and chromatographic resolution of sterols and oxysterols (25), but the method often requires sample derivatization with Girard-P hydrazine (16) and picolinyl esterification (17) to improve ionization efficiencies. In GC-MS with electron-impact ionization, the TMS derivative of steroids provides less structural information than its TMS analog, which is better for screening purposes due to the absence of extensive fragmentation (26). Initially, 7α-OHC was analyzed, but it formed two chromatographic peaks (at 4.73 min and 4.96 min) with similar mass spectra (data not shown), while 7β-OHC resulted in a single chromatographic peak. The first peak of 7α-OHC overlapped with the high concentration of cholesterol peak in serum samples, and the second (with approximately one-third the height intensity of the first peak) was coeluted with desmosterol, which is successfully quantified at *m/z* 343 in SIM analysis. Therefore, 7α-OHC was not chosen as the analyte. In the present TMS derivatization with a mixture of *N*-methyl-*N*-trimethylsilyltrifluoroacetamide (MSTFA)/NH_4_I/DTE, the same analytical phenomena were observed in 7α- and 7β-hydroxy-dehydroepiandrosterone (7α-/7β-OH-DHEA; data not shown). Further analytical study would be accomplished in 5-ene-7-hydroxylated steroid molecules.

If both a double bond and a carbonyl group are present in a structure, the double bond can be hydrogenated, with or without leaving the carbonyl intact (which depends on the reducing agent). In this study, the hydrogenation of a double bond at C-5 and C-6 may give an isomeric hydrogen at the C-5 position, which is different with biosynthetic 7-hydroxylation. In contrast to the conventional GC-MS technique using a fused-silica capillary column with a long runtime (over 60 min) (19), HTGC-MS with a thermally stable stainless steel capillary column is described as an alternative technique for the analysis of lipophilic compounds (21). In our previous studies, it successfully achieved good chromatographic properties for the analysis of lipid molecules including cholesterols (23, 27).

The sample preparation techniques were also investigated for the removal of endogenous matrix components from lipid-rich samples (28–30). In particular, phospholipids are extremely abundant in biological samples and are known to cause significant matrix effects. The H-PPT uses the principle of Lewis acid-base interactions between the zirconia-coated particles used as a sorbent and the phospholipid, which provides simple, rapid, and selective removal of interference (20). In this study, the simultaneous analysis of 18 sterols was carried out using HTGC-MS combined with H-PPT. As the results indicate, all of the analytes in serum were successfully detected without any matrix effects, and there was good detectability. Moreover, the validated method showed good linearity and acceptable LOQ from 20 μl of human serum.

In general, cardiovascular events are more common in men than in women because of the protective effects of estrogens prior to menopause. However, the risk of cardiovascular disease significantly increases and becomes parallel to that for males in post-menopausal females. Lipoprotein metabolism also shows sex differences (31): cardiovascular events are less likely in pre-menopausal women than in men because of higher levels of high density lipoproteins (HDLs) and lower levels of low density lipoproteins (LDLs), triglycerides, and total cholesterol. To understand sex differences in cardiovascular events, risk factors such as blood pressure, body mass index, smoking rate, and levels of cholesterol, HDLs, and LDLs have been investigated (32–34). Among these risk factors, the level of HDL cholesterol and the smoking rate explain a substantial part of the sex difference. In this study, cholesterol metabolism was evaluated in male and female patients with vasospastic angina, and sex-related differences in cholesterol synthetic pathways from desmosterol, which are indicative of the activities of 24-reductase, were observed (Fig. 3). Because the precursor of cholesterol can be generated in response to cholesterol loading (35), the results could be considered an increased accumulation of cholesterol in females.

In conclusion, the present HTGC-MS-based quantitative cholesterol signatures have been conducted with H-PPT purification and GC separation through a HTGC column separation. Although cholesterols are presented in most mammalian bodies, the proportion of esters formed to free cholesterol varies markedly. Serum cholesterol is mainly found in its ester forms (over 80%), but no cholesterol esters are present in the brain and other nervous tissues. An understanding of cholesterol transport and CE metabolism is crucial in cardiovascular physiology, the devised method here was aimed at individual quantification of cholesterol and its esters, as well as oxidation metabolites, to evaluate the different physiological responses of cholesterol. This method was successfully applied to detect sex differences in vasospastic angina. Therefore, this method can be used to understand cholesterol metabolism, and it may provide useful information about physiological differences between males and females.

## Supplementary Material

Supplemental Data
