# Supplementary material for: High-temperature GC-MS-based serum cholesterol signatures may reveal sex differences in vasospastic angina
Source: J Lipid Res. 2014 Jan;55(1):155–62. doi: 10.1194/jlr.D040790 (PMC3927468; doi:10.1194/jlr.D040790)
Supplement: Supplemental Data [file supp_55_1_155__index.html]

High-temperature GC-MS-based serum cholesterol signatures may reveal sex differences in vasospastic angina — High-temperature GC-MS-based serum cholesterol signatures may reveal sex differences in vasospastic angina — Supplemental Data 

# High-temperature GC-MS-based serum cholesterol signatures may reveal sex differences in vasospastic angina

## Supplemental Data

**Files in this Data Supplement:**

- Supplemental Tables 1/2 and Figure 1 - Results of the method validation and the selected-ion chromatograms of analytes detected in the serum sample
