# Supplementary material for: High-temperature GC-MS-based serum cholesterol signatures may reveal sex differences in vasospastic angina
Source: J Lipid Res. 2014 Jan;55(1):155–62. doi: 10.1194/jlr.D040790 (PMC3927468; doi:10.1194/jlr.D040790)

Supplemental Materials

for

High-temperature GC-MS–based serum cholesterol signatures may reveal sex differences in vasospastic angina

Hyun-Hwa Son[*], Ju-Yeon Moon[*], Hong Seog Seo[†,1], Hyun Hee Kim[†], Bong Chul Chung[*], and Man Ho Choi[*,1]

[*]Future Convergence Research Division, Korea Institute of Science and Technology, Seoul 136-791, Korea;

[†]Cardiovascular Center, Korea University Guro Hospital, Seoul 152-703, Korea

[*]Corresponding Authors: **Man Ho Choi, Ph. D.**

Future Convergence Research Division, KIST

39-1 Hawolkok-dong, Seoul 136-791, Korea

(Tel) +82-2-958-5081 (Fax) +82-2-958-5059

(e-mail) mh_choi@kist.re.kr

Hong Seo Seo, M.D., Ph.D.

Cardiovascular Center, Korea University Guro Hospital

80 Guro-dong, Seoul 152-703, Korea

(Tel) +82-2-2626-3018 (Fax) +82-2-863-1109

(e-mail) mdhsseo@unitel.co.kr

The supplemental materials contain the results of the method validation and the selected-ion chromatograms of analytes detected in the serum sample.

Supplemental Table 1. Validation results of the overall method for the intra- and inter-day assays in serum

Compounds	Calibration range[a] (μg/mL)	Calibration equations	Linearity (r^2)	Recovery[b] (%)	Intra-day (n = 5)		Inter-day (n = 5)	
					CV[c] (%)	Accuracy[d] (%)	CV (%)	Accuracy (%)
Cholesterol	10.00 ~ 1000	y= 0.0175x+0.3082	0.9941	100.6	5.5	98.2	4.2	98.6
**Cholesteryl esters**								
Cholesteryl laurate	0.20 ~ 20	y= 0.0455x-0.0254	0.9896	64.0	4.8	96.4	8.6	98.2
Cholesteryl myristate	2.00 ~ 100	y= 0.0140x-0.0564	0.9808	49.8	5.6	95.0	6.4	97.1
Cholesteryl palmitate	5.00 ~ 1000	y= 0.0419x-0.3874	0.9925	38.0	8.9	104.3	5.7	88.2
Cholesteryl oleate & linoleate	10.00 ~ 1000	y= 0.0342x+0.1354	0.9870	54.6	9.8	75.9	9.3	76.5
Cholesteryl stearate	2.00 ~ 1000	y= 0.0048x-0.0080	0.9987	26.1	1.1	101.1	4.3	102.4
**Cholesterol precursors**								
Desmosterol	0.10 ~ 5	y= 0.0017x-0.1736	0.9961	90.8	6.6	103.9	4.4	111.7
Lathosterol	0.02 ~ 5	y= 0.0018x-0.1055	0.9971	91.8	4.9	125.1	5.3	122.9
Lanosterol	0.02 ~ 5	y= 0.0019x-0.1443	0.9955	91.6	5.1	123.1	4.6	115.7
**Hydroxycholesterols**								
7-Ketocholesterol	0.01 ~ 1	y= 0.0074x-0.0173	0.9976	99.8	5.5	99.0	5.0	102.0
7β-Hydroxycholesterol	0.01 ~ 1	y= 0.0097x-0.0143	0.9977	83.8	7.5	105.3	5.5	103.1
4β-Hydroxycholesterol	0.02 ~ 1	y= 0.0017x-0.0008	0.9983	88.4	4.0	90.5	4.7	98.1
20α-Hydroxycholesterol	0.01 ~ 1	y= 0.0106x-0.0825	0.9982	129.3	2.9	96.2	3.5	93.8
27-Hydroxycholesterol	0.02 ~ 1	y= 0.0007x+0.0167	0.9977	99.0	4.3	98.3	5.0	99.2
24-Hydroxycholesterol	0.02 ~ 1	y= 0.0014x+0.0015	0.9987	100.9	4.2	95.0	4.9	96.4
19-Hydroxycholesterol	0.02 ~ 1	y= 0.0033x-0.0305	0.9969	100.3	3.2	100.0	3.4	99.2
25-Hydroxycholesterol	0.10 ~ 5	y= 0.0003x-0.0331	0.9966	103.3	2.6	82.6	3.0	97.1

CV, coefficient of variance.
[a]The calibration range was included the limit of quantification and covered the serum concentrations measured.
[b]Absolute recoveries were calculated by comparing peak height ratios of extracted samples and those of standard samples without sample preparation using 3 different quality control (QC) samples in triplicate.
[c, d]Precision and accuracy are expressed as the mean values of data obtained from 3 QC samples through intra- and inter-day assays.

Supplemental Table 2. The results of the stability tests for working solution, short-term storage, freeze-thaw cycles, and post preparations

Compounds	Working solution (%)			Short-term storage (%)			Freeze-thaw cycles (%)			Post-preparative stability (%)					
										Re-injection after 12 hr			Re-injection after 24 hr		
	Low	Med	High	Low	Med	High	Low	Med	High	Low	Med	High	Low	Med	High
Cholesterol	91.3	96.3	100.2	84.3	92.1	82.8	91.4	97.6	100.9	91.7	99.3	101.4	100.8	97.6	97.7
**Cholesteryl esters**															
Cholesteryl laurate	96.1	93.1	94.5	35.0	24.6	36.0	50.6	52.9	57.7	101.7	101.9	110.1	99.9	103.0	105.9
Cholesteryl myristate	91.7	97.8	94.4	25.7	35.4	47.7	63.6	62.3	74.9	102.0	103.3	100.8	98.9	97.2	100.3
Cholesteryl palmitate	90.3	88.5	91.5	44.9	64.7	61.0	77.6	107.5	106.9	102.1	100.9	86.5	101.9	100.1	85.5
Cholesteryl oleate & linoleate	91.5	87.9	91.1	49.3	69.8	71.4	66.2	113.3	114.1	101.2	98.9	88.3	100.7	97.2	86.6
Cholesteryl stearate	96.1	97.5	98.8	57.2	73.7	82.9	27.4	31.8	89.9	111.4	105.9	101.1	110.6	110.2	103.7
**Cholesterol precursors**															
Desmosterol	108.8	97.0	99.1	90.6	91.8	92.9	95.7	98.4	95.0	102.9	96.5	94.6	100.6	101.1	101.1
Lathosterol	95.0	98.8	100.2	101.6	92.3	92.8	85.7	104.2	98.3	99.1	103.9	95.0	91.4	102.9	100.5
Lanosterol	98.7	94.6	98.2	84.9	80.9	88.9	89.7	91.7	95.3	98.3	99.1	100.3	97.5	99.6	97.9
**Hydroxycholesterols**															
7-Ketocholesterol	99.7	100.5	97.9	91.9	95.2	108.9	97.2	100.7	95.5	101.1	100.0	97.9	100.2	95.9	97.6
7β-Hydroxycholesterol	100.2	99.8	100.3	90.5	100.5	109.7	97.2	100.7	94.7	104.8	97.3	97.9	101.7	96.1	97.1
4β-Hydroxycholesterol	98.5	105.2	96.2	92.8	99.5	103.6	104.5	100.8	96.1	109.3	99.5	99.3	105.5	97.4	97.5
20α-Hydroxycholesterol	105.5	106.5	111.0	90.3	102.4	106.1	91.3	104.9	106.3	98.0	98.8	102.1	108.7	102.8	103.8
27-Hydroxycholesterol	97.0	97.1	100.1	95.4	101.1	99.3	105.2	101.9	97.4	98.7	98.4	98.7	99.4	96.6	100.9
24S-Hydroxycholesterol	97.4	100.7	98.0	96.9	99.4	98.9	92.9	98.5	98.9	98.7	94.7	100.0	98.8	93.5	99.5
19-Hydroxycholesterol	100.5	97.8	101.6	99.3	108.1	101.4	93.5	102.5	102.3	94.4	95.5	102.1	94.3	93.3	99.0
25-Hydroxycholesterol	97.9	101.2	96.3	97.6	101.1	98.0	98.7	100.2	102.8	93.9	99.3	100.6	97.5	102.1	99.4

The stability was tested by comparing the results of the samples analyzed before and after being exposed to the conditions for the stability assessment at three different concentrations in triplicates.

Supplemental Fig. 1. The detection windows of selected-ion chromatograms of analytes detected in the serum sample.

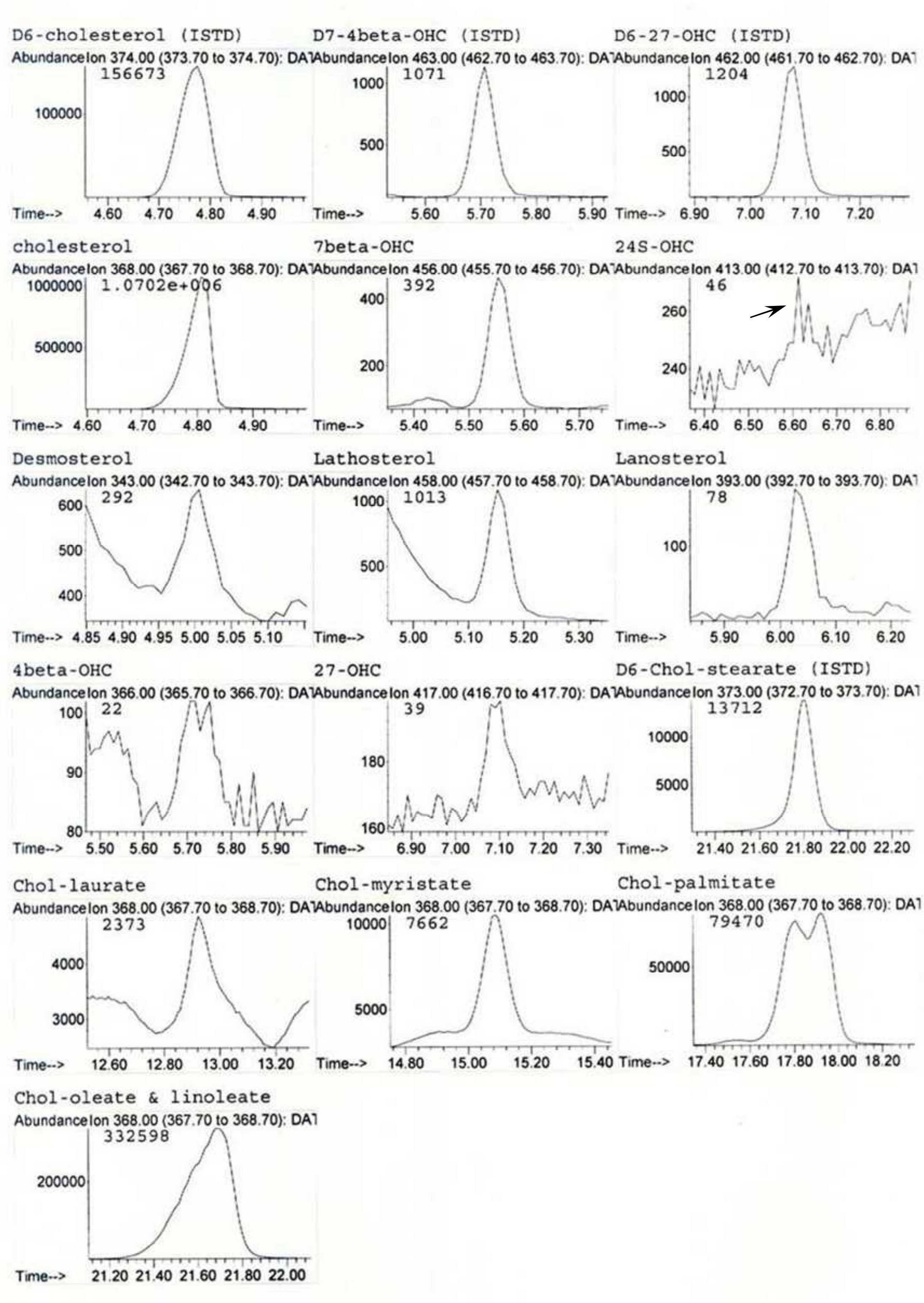

Supplement: Supplemental Data [file supp_D040790_jlr.D040790-1.pdf]
